# Genome-wide identification, characterization, and expression analysis of m6A readers-YTH domain-containing genes in alfalfa

**DOI:** 10.1186/s12864-023-09926-w

**Published:** 2024-01-02

**Authors:** Shugao Fan, Xiao Xu, Jianmin Chen, Yanling Yin, Ying Zhao

**Affiliations:** School of Resources and Environmental Engineering, Ludong University, Yantai, China

**Keywords:** Alfalfa, YTH domain, Gene family, m6A methylation, Salt, Cold stress

## Abstract

Eukaryotic messenger RNAs (mRNAs) are often modified with methyl groups at the N6 position of adenosine (m6A), and these changes are interpreted by YTH domain-containing proteins to regulate the metabolism of m6A-modified mRNAs. Although alfalfa (*Medicago sativa*) is an established model organism for forage development, the understanding of YTH proteins in alfalfa is still limited. In the present investigation, 53 putative YTH genes, each encoding a YT521 domain-containing protein, were identified within the alfalfa genome. These genes were categorized into two subfamilies: *YTHDF* (49 members) and *YTHDC* (four members). Each subfamily demonstrates analogous motif distributions and domain architectures. Specifically, proteins encoded by *MsYTHDF* genes incorporate a single domain structure, while those corresponding to *MsYTH5, 8, 12, 16* who are identified as members of the *MsYTHDC* subfamily, exhibit CCCH-type zinc finger repeats at their N-termini. It is also observed that the predicted aromatic cage pocket that binds the m6A residue of MsYTHDC consists of a sequence of two tryptophan residues and one tyrosine residue (WWY). Conversely, in MsYTHDF, the binding pocket comprises two highly conserved tryptophan residues and either one tryptophan residue (WWW) or tyrosine residue (WWY) in MsYTHDF.

Through comparative analysis of qRT-PCR data, we observed distinct expression patterns in specific genes under abiotic stress, indicating their potential regulatory roles. Notably, five genes (MsYTH2, 14, 26, 27, 48) consistently exhibit upregulation, and two genes (MsYTH33, 35) are downregulated in response to both cold and salt stress. This suggests a common mechanism among these YTH proteins in response to various abiotic stressors in alfalfa. Further, integrating qRT-PCR with RNA-seq data revealed that MsYTH2, MsYTH14, and MsYTH16 are highly expressed in leaves at various development stages, underscoring their potential roles in regulating the growth of these plant parts. The obtained findings shed further light on the biological functions of *MsYTH* genes and may aid in the selection of suitable candidate genes for future genetic enhancement endeavors aimed at improving salt and cold tolerance in alfalfa.

**Supplementary Information:**

The online version contains supplementary material available at 10.1186/s12864-023-09926-w.

## Introduction

Posttranscriptional regulation is essential for plant growth, development, and stress response [13]. RNA-binding proteins are key players in these processes, either through direct interaction with RNA or by indirectly modulating other regulatory factors [27]. Among the various post-transcriptional modifications, N6-methyladenosine (m6A) is the most prevalent and influential modification in eukaryotic mRNAs and noncoding RNAs which is deposited by the writer complex (MTA-MTB-FIP37-VIR-HAKAI in plants) and can be deleted by erasers (ALKBH proteins) [19, 55]. The processes of m6A methylation and demethylation illustrate the reversibility of m6A modification, leading to dynamic changes of m6A levels in vivo [52]. Reader proteins can recognize the m6A mark and participate in a wide range of processes involving RNA metabolism, such as splicing, translation efficiency, stability, alternative polyadenylation, structure, nuclear export, and also exert epigenetic effects on noncoding RNAs [3, 6, 33, 41, 42, 49, 57]. According to Patil et al. [38] and Reichel et al. [41], reader proteins are responsible for determining the fate of m6A-modified mRNA. It is therefore essential to identify the m6A readers to unravel the mechanism by which m6A functions in cells.

The YT521-B homology (YTH) domain was first identified by matching known protein sequences with the splicing factor YT521-B, which is involved in RNA processing and interacts with other proteins [25, 53]. The YTH domain serves as the module for recognizing m6A in a methylation-dependent manner. There are five known YTH domain-containing proteins in humans, including YTHDC1, YTHDC2, YTHDF1, YTHDF2, and YTHDF3. The YTH domain-containing proteins extensively participate in post-transcriptional regulation. They also influence splicing, translation, localization, and RNA lifetime. Those processes involve targeting different complexes to specific sites through direct binding to m6A [50, 54]. The crystal structures of YTHDC2 and YTHDC1 domains have been compared, revealing a conserved hydrophobic pocket and positively charged surface for recognizing m6A RNA [30]. Similarly, YTHDF1, YTHDF2, and YTHDF3 also possess aromatic cages and specific residues for m6A recognition, while a basic patch contributes to RNA backbone binding [44]. In turn, the conservation of these amino acid residues across evolution and their analysis in Arabidopsis ECT protein mutants suggest that these residues likely have the same function in plant YTH proteins [4]. Scutenaire et al. [43] performed a systematic evolutionary analysis of YTH domains in Viridiplantae. They demonstrated that vascular plants display both YTHDF- and YTHDC-type motifs. These motifs carry the amino acids necessary for RNA binding and to accommodate m6A, and are predicted to adopt the same structural fold as YTH domains in animals and yeast.

It is of particular interest that plants possess a higher number of YTH domain proteins compared to other organisms, as suggested by Shi et al. [44]. The Arabidopsis genome harbors 13 YTH domain proteins which can be categorized into two groups. The larger group, with 11 YTHDF proteins, is classified as EVOLUTIONARY CONSERVED C-TERMINAL REGION proteins, owing to their conserved C-terminal region. The smaller group consists of 2 YTHDC proteins, *At4g11970* (also known as *AtYTH11*) and *CPSF30* (also known as *AtYTH03*) as reported by Scutenaire et al. [43] and Arribas-Hernández et al. [4].

A recent study by Govindan et al. [16] demonstrated that the increased RNA methylation, specifically the m6A modification, significantly contributes to the regulation of cold tolerance. Under salt stress, m6A is dynamically deposited on transcripts, stabilizing the genes that encode proteins involved in salt and osmotic stress responses [2]. In rice, cadmium treatment results in differential m6A modifications in thousands of transcripts in the root, implying that m6A might be linked to abnormal root development caused by cadmium stress [9]. In wheat, genes encoding the m6A reader protein TaYTHs display noticeable expression changes in response to abiotic stresses like water and drought. Additionally, salt stress in sweet sorghum leads to significant changes in the m6A methylome, increasing m6A modification and mRNA stability of salt-resistance genes, which in turn positively regulates tolerance to salt stress [56]. Significant alterations in the m6A methylome profile, along with its correlation with mRNA abundance, have been identified in pak choi, tomato, and apple leaf [28, 32, 51]. These findings suggest that m6A modification also plays a role in modulating crops’ responses to temperature and humidity-induced stresses.

While significant research has been conducted on the YTH domain proteins of several plant species, our understanding of the YTH family members in forage species like alfalfa remains limited. Alfalfa is a highly valuable feed crop due to its high protein content and capacity to fix atmospheric nitrogen. A comprehensive genome-wide understanding of the YTH gene family in alfalfa is crucial for deciphering the molecular processes underpinning RNA modification and gene regulation in this important crop. Therefore, this study aims to identify and analyze the YTH gene family in alfalfa using a comprehensive genome-wide approach. By employing bioinformatics methods, we analyzed the whole genome sequence of alfalfa to systematically identify YTH genes. The discovered YTH genes were further investigated for their structural characteristics, conserved domains, evolutionary linkages, and expression patterns under various organs and abiotic stresses. The findings from this study could pave the way for future genetic engineering and breeding initiatives focused on enhancing alfalfa quality and yield by targeting the identified YTH genes.

## Material and methods

### Plant growth and sample collection

The alfalfa cultivar used in this study for qRT-PCR analysis was Xinjiang Daye, aligning with the genome data for this investigation. The Xinjiang Daye seeds were provided by Dr. Rui Dong (Guizhou University). For each treatment, three seeds were sown in each plastic pot, and this process was replicated three times. Subsequently, from each pot, a seedling was selected based on identical growth characteristics observed within that specific pot, while the other two seedlings from the same pot were discarded. The plants were grown with a mixture of soil (vermiculite/humus = 1:1) under a 16/8 h day/night cycle, 25°/20 °C day/night temperatures, and a light intensity of 300 mol m^−2^ s^−1^. To validate the gene expression patterns across different plant organs, which are stem, root, leaf and flower, samples were collected from 8-week-old plants during the flowering stage. To assess gene expression under cold and salt treatments, seedlings were subjected to cold treatment at 4 °C and salinity treatment with 200 mM NaCl for 6 h. Subsequently, leaf samples were collected for gene expression analysis. Control groups were implemented for each treatment, involving seedlings maintained under normal growth conditions at 25 °C (day) / 20 °C (night), without exposure to salinity stress. Upon harvest, all samples were promptly frozen in liquid nitrogen and stored at -80 °C until use.

### In-silico identification of the YTH gene family

The genome and annotation data for the alfalfa cultivar “Xinjiang daye” were retrieved from the Figshare data repository (https://figshare.com/projects/whole_genome_sequencing_and_assembly_of_Medicago_sativa/66380) [8]. To identify YTH domain homologs in alfalfa, the Hidden Markov model of the HMMER 3.0 software was used (PF04146). The *Arabidopsis thaliana* YTH members identified by Li et al. [24] were retrieved from The Arabidopsis Information Resource (http://www.arabidopsis.org/index.jsp) and were used as queries in a BLASTp search against all the protein sequences of the alfalfa genome with an E-value threshold of 1e^−5^. The genes identified using both approaches were compiled, and duplicate genes were manually removed. The presence of the YTH domain was verified using the CDD (https://www.ncbi.nlm.nih.gov/cdd) and Interpro (https://www.ebi.ac.uk/interpro) databases [39, 47]. Sequences that lacked the whole YTH domain were eliminated. The discovered genes were named according to their chromosomal position. The ProtParam tool (http://web.expasy.org/protparam/) was used to determine the number of amino acids (AA) characteristics, molecular weights (MW), and isoelectric points (pIs), grand average of hydropathy (GRAVY) and Cell-PLoc (http://www.csbio.sjtu.edu.cn/bioinf/Cell-PLoc/) was used to estimate the subcellular localizations [10, 15].

### Phylogeny and chromosomal location

Sequences of YTH protein in Arabidopsis and alfalfa were aligned using Clustal X with default parameters [21]. The phylogenetic tree of YTH family members in alfalfa and Arabidopsis was generated using MEGA X software. The unrooted phylogenetic tree was built using the Neighbor-joining (NJ) technique with a bootstrap value of 1000 iterations, and the phylogenetic trees were drawn using the iTOL (Interactive Tree of Life) web program (http://itol.embl.de/) [23]. The chromosomal location of all discovered genes was determined from the *M. sativa* genomic annotation file GFF3 and named based on their locations on chromosome. We plotted the chromosomal distribution of MsYTH genes using the TBtools program [8].

### Gene duplication events and gene selection pattern

Gene duplication events in MsYTH genes were detected and identified utilizing collinear scanning toolkits (MCScanX) with an e-value of 10^–5^ [48]. Non-synonymous (Ka) / synonymous (Ks) ratios for each gene pair were calculated using the DnaSP5 to analyze the evolutionary constraints of the MsYTH genes [26].

### Gene structure and functional prediction

MEME (http://meme-suite.org/tools/meme) was used to identify conserved motifs in MsYTH family members and the WebLogo tool was utilized to construct the theme Logos [5, 11]. Using the TBtools program, the exon–intron structure of MsYTH genes was visualized by constructing a gene structure based on the CDS and the associated full-length sequence. The MsYTH genes’ upstream regions (-1500 bp) were explored for regulatory elements. Plantcare (http://bioinformatics.psb.ugent.be/webtools/plantcare/html/) was used to identify and visualize the cis-acting regulatory elements in the promoter [22]. ESPrint3.0 (https://espript.ibcp.fr/ESPript/cgi-bin/ESPript.cgi) was used to analyze the protein domain. The 3D structure of MsYTH proteins was constructed using AlphaFold2 v1.5.1 with the default parameters [34].

### Expression pattern of MsYTH in different organs by RNA-seq

The expression of MsYTH members was examined using RNA-seq data from various organs, obtained from alfalfa database LegumeIP V3 (https://www.zhaolab.org/LegumeIP) [12]. Six different organs of CADL alfalfa (Cultivated Alfalfa at the Diploid Level), namely root, nodule, leaf, flower, pre-elongated stem, elongated stem, were included in the analysis. Three replicates were performed for each organ. The FPKM values obtained from the RNA-seq were normalized using the log_10_(FPKM) treatment, and a heatmap was generated using HemI software [35].

### Validation of MsYTH genes expression in various organs and under abiotic stress using qRT-PCR

For further validation, the expression of the MsYTHs genes was analyzed using quantitative real-time polymerase chain reaction (qRT-PCR). Total RNA was extracted from the harvested samples using the Trizol reagent according to the manufacturer’s instructions. The quality and quantity of RNA were assessed using a NanoDropTM UV spectrophotometer (Thermo Fisher Scientific, Lenexa, KS, USA). To ensure the accuracy of the analysis, genomic DNA contamination was eliminated, and first-strand cDNA synthesis was performed using the PrimeScriptTM RT reagent Kit with gDNA Eraser on 1 µg of total RNA (Takara, Japan). The qRT-PCR study was conducted using a CFX96 TouchTM Real-Time PCR Detection System (Bio-Rad, USA). The housekeeping gene EF-1α was used as a reference. Primer details are provided in Table S2. The relative expression values for YTH family genes were calculated using the 2^−∆∆Ct^ method, as described by Livak and Schmittgen [29].

Relative gene expression levels were assessed after normalization against the expression levels of the housekeeping gene. Each conditions’s value represents the mean expression level, and error bars indicate the standard deviation (SD) calculated from three independent biological replicates. Statistical significance between different groups was determined by one-way ANOVA with posterior Duncan test (*p* < 0.05), with different lowercase letters marking significantly different groups.

## Results

### Identification of the YTH gene family in alfalfa

A total of 53 putative YTH genes were discovered and designated by their chromosomal position. Table 1 lists gene IDs, chromosomal locations, chromosome start, and end positions, amino acid counts, molecular weights, pIs, GRAVY, and potential subcellular localizations of proteins. The sequences of all the MsYTH proteins are provided in Supplemental Table S1. Members of the YTH family are distributed unevenly on alfalfa chromosomes. The number of composing amino acid residues was variable as well. This variability ranged from the shortest/smallest being 169 amino acids to the longest/biggest being 853 amino acids. The MW ranges from 18.83 kDa (MsYTH30) to 96.61 kDa (MsYTH13). The pIs vary from 4.97 (MsYTH17) to 9.57 (MsYTH43). The majority of YTH proteins are likely to be found in the nucleus, with nine members predicted to be located in both the cell wall and nucleus, one likely in the cell wall, and one predicted in the chloroplast.

**Table 1:** Characteristics of YTH gene family identified in alfalfa

Name	chr	Start	End	Strand	AA^a^	MW^b^	pI^c^	GRAVY^d^	Localization
MsYTH1	chr2.1	32,932,610	32,936,663	-	594	65.96	6.29	-0.728	Cell wall. Nucleus
MsYTH2	chr2.1	32,946,789	32,950,076	-	553	61.46	6.9	-0.755	Nucleus
MsYTH3	chr2.1	32,976,806	32,980,860	+	594	65.97	6.29	-0.728	Cell wall. Nucleus
MsYTH4	chr2.1	59,723,529	59,727,525	-	695	76.63	6.45	-0.636	Cell wall. Nucleus
MsYTH5	chr2.1	61,439,122	61,449,530	-	683	75.27	6.31	-0.946	Nucleus
MsYTH6	chr2.1	75,968,015	75,978,538	+	842	95.29	8.68	-0.605	Nucleus
MsYTH7	chr2.2	56,045,788	56,049,775	-	695	76.58	6.63	-0.625	Nucleus
MsYTH8	chr2.2	58,268,689	58,278,974	-	683	75.18	6.28	-0.946	Nucleus
MsYTH9	chr2.2	73,598,530	73,600,767	+	368	42.6	9.33	-0.837	Nucleus
MsYTH10	chr2.3	31,065,853	31,069,907	-	594	65.96	6.29	-0.728	Cell wall. Nucleus
MsYTH11	chr2.3	57,877,905	57,881,747	-	691	76.2	6.63	-0.0636	Cell wall. Nucleus
MsYTH12	chr2.3	60,011,762	60,022,164	-	683	75.27	6.31	-0.946	Nucleus
MsYTH13	chr2.3	76,007,416	76,018,004	+	853	96.61	8.06	-0.576	Nucleus
MsYTH14	chr2.4	30,598,272	30,602,194	-	583	64.8	6.37	-0.749	Cell wall. Nucleus
MsYTH15	chr2.4	57,831,563	57,835,405	-	691	76.2	6.63	-0.0636	Cell wall. Nucleus
MsYTH16	chr2.4	60,185,027	60,195,458	-	683	75.25	6.22	-0.943	Nucleus
MsYTH17	chr2.4	76,326,146	76,327,374	+	320	35.28	4.97	-0.624	Nucleus
MsYTH18	chr2.4	76,356,848	76,359,085	-	368	42.63	9.32	-0.832	Nucleus
MsYTH19	chr3.4	24,081,850	24,082,422	+	190	21.39	8.81	-1.093	Nucleus
MsYTH20	chr4.1	12,219,900	12,223,122	+	670	73.39	6.07	-0.804	Nucleus
MsYTH21	chr4.1	89,468,284	89,477,638	-	641	70.23	5.73	-0.662	Nucleus
MsYTH22	chr4.2	74,684,133	74,686,905	-	337	37.1	5.17	-0.715	Nucleus
MsYTH23	chr4.3	12,762,941	12,766,198	+	670	73.41	6.07	-0.818	Nucleus
MsYTH24	chr4.3	12,839,747	12,843,004	+	670	73.36	6.18	-0.808	Nucleus
MsYTH25	chr4.3	89,750,461	89,760,129	-	642	70.35	5.73	-0.658	Nucleus
MsYTH26	chr4.3	89,793,760	89,803,403	-	642	70.32	5.73	-0.662	Nucleus
MsYTH27	chr4.4	12,725,637	12,728,824	+	670	73.42	6.07	-0.807	Nucleus
MsYTH28	chr6.1	11,307,171	11,310,415	+	640	70.17	5.94	-0.866	Nucleus
MsYTH29	chr6.2	20,909,412	20,912,592	+	640	70.23	5.94	-0.867	Nucleus
MsYTH30	chr6.2	81,112,371	81,113,150	-	169	18.83	5.35	-1.038	Nucleus
MsYTH31	chr6.3	20,789,008	20,792,252	+	640	70.17	5.94	-0.866	Nucleus
MsYTH32	chr6.3	50,095,718	50,096,497	-	184	20.75	8.5	-1.081	Nucleus
MsYTH33	chr6.3	72,348,467	72,349,952	+	330	36.54	5.46	-0.776	Nucleus
MsYTH34	chr6.4	3,991,336	3,994,517	+	639	70.06	6.17	-0.863	Nucleus
MsYTH35	chr6.4	30,939,582	30,942,120	-	246	28.22	9.31	-1.156	Nucleus
MsYTH36	chr7.1	19,538,558	19,543,168	+	658	71.67	8.22	-0.712	Nucleus
MsYTH37	chr7.1	66,356,530	66,358,127	-	778	89.53	9.22	-0.826	Nucleus
MsYTH38	chr7.2	22,246,375	22,250,996	+	659	71.76	8.22	-0.717	Nucleus
MsYTH39	chr7.2	67,774,919	67,777,830	-	388	44.72	9.52	-0.857	Nucleus
MsYTH40	chr7.3	23,056,195	23,060,813	+	683	75.27	6.31	-0.946	Nucleus
MsYTH41	chr7.3	68,900,717	68,903,501	-	236	26.85	9.02	-0.608	Nucleus
MsYTH42	chr7.4	21,834,081	21,838,717	+	660	72.08	8.22	-0.708	Nucleus
MsYTH43	chr7.4	70,311,054	70,314,017	-	322	36.74	9.57	-1.018	Nucleus
MsYTH44	chr8.1	7,747,835	7,751,049	+	652	72.28	6.43	-0.617	Nucleus
MsYTH45	chr8.1	40,044,024	40,045,704	-	330	36.54	5.46	-0.776	Nucleus
MsYTH46	chr8.2	10,496,232	10,499,446	+	652	72.29	6.43	-0.616	Nucleus
MsYTH47	chr8.3	1,971,108	1,972,788	+	330	36.44	5.46	-0.753	Nucleus
MsYTH48	chr8.3	5,633,457	5,636,671	+	652	72.25	6.43	-0.619	Cell wall. Nucleus
MsYTH49	chr8.4	62,764,812	62,765,763	-	211	23.68	6.83	-0.854	Cell wall
MsYTH50	14,996	11,267	14,485	+	403	45.9	7.63	-0.428	Chloroplast
MsYTH51	14,997	6917	10,969	+	582	64.79	6.18	-0.746	Cell wall. Nucleus
MsYTH52	23,421	5234	6914	-	320	35.34	5.31	-0.752	Nucleus
MsYTH53	chr2.1	32,919,537	32,923,591	-	594	65.96	6.29	-0.728	Cell wall. Nucleus

^a^*AA* Number of amino acids

^b^*MW* Molecular weights(kDa)

^c^*pI* Isoelectric points

^d^*GRAVY* Grand average of hydropathy

### Phylogenetic analysis and conserved domain of YTH members

To gain insights into the evolutionary dynamics of YTH domain-containing proteins in alfalfa, an unrooted phylogenetic tree was constructed using a dataset comprising the 53 sequences from alfalfa and 13 YTH protein sequences from Arabidopsis retrieved from the TAIR database.

**Fig. 1:**
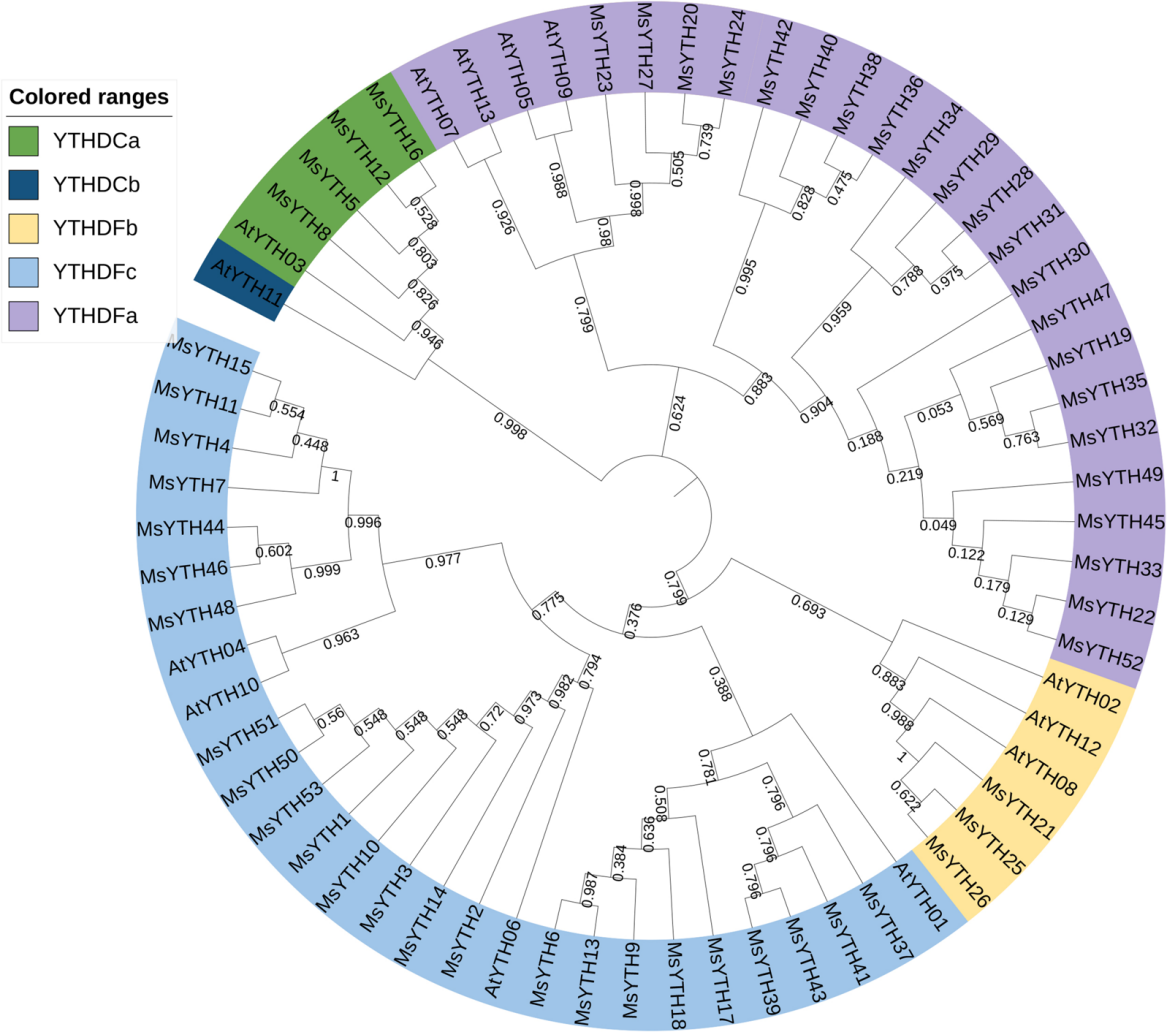
Phylogenetic relationship between YTH proteins identified in alfalfa and Arabidopsis. The phylogenetic tree was constructed using the Neighbor-jointing method with 1000 bootstrap replications. The five subclades of YTHDCa, YTHDCb, YTHDFa, YTHDFb and YTHDFc are labeled with different colors

Results showed that MsYTH proteins were divided into two distinct clades: YTHDF and YTHDC subfamilies (Fig. 1). According to the evolutionary tree, no close relatives were found in Arabidopsis and Alfalfa in any clade. There are 22, 3 and 24 protein sequences in the further subclades YTHDFa, YTHDFb and YTHDFc, respectively. The YTHDC subfamily was divided into two subclades, YTHDCa, which included four alfalfa sequences, and YTHDCb. Notably, the YTHDCb subclade did not contain any MsYTH protein, indicating a possible evolutionary divergence between alfalfa and other species in the YTHDC subfamily. Together, these results indicate that the YTH family proteins were highly conserved in alfalfa, and that there was a slight evolutionary divergence in the subfamily or subclade.

**Fig. 2:**
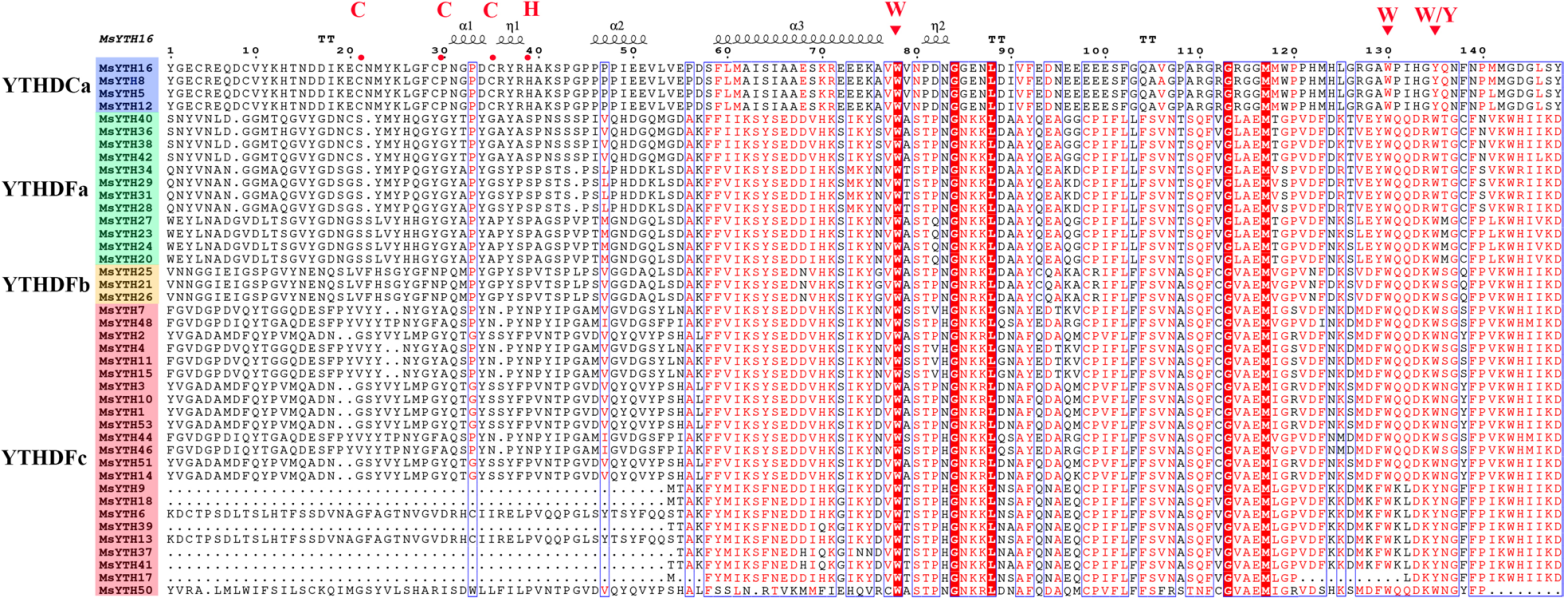
Multiple sequence alignment showing the conserved domains of the alfalfa YTH protein sequences. The secondary structural elements predicted with AlphaFold DB model of A0A3Q7Y049_CICAR are shown above. The red triangles represent the key functional residues WWW/WWY, and the red dot represent the key functional residues CCC-H within the YTHDC subfamily

Additional multiple sequence alignment of YTH proteins showed that many functional sites were conserved and displayed a conserved aromatic cage (Fig. 2), suggesting that MsYTHs might have a similar m6A read mechanism to human YTH proteins especially in subfamily YTHDFs. Our research uncovered that the aromatic cage that binds the m6A residue within MsYTHDC proteins are highly conserved and consist of two tryptophan residues and one tyrosine residue (WWY). In contrast, the aromatic cage in MsYTHDFa and MsYTHDFb proteins are composed of three conserved tryptophan residues (WWW), whereas in MsYTHDFc, it consists of two highly conserved tryptophan residues and either one tryptophan residue (WWW) or tyrosine (WWY). Specifically, proteins encoded by MsYTHDF genes incorporate a single domain structure, while those corresponding to MsYTH5, 8, 12, 16 identified as members of the MsYTHDC subfamily, exhibit CCCH-type zinc finger repeats at their N-termini.

### Gene chemical structure and conserved motif analysis

**Fig. 3:**
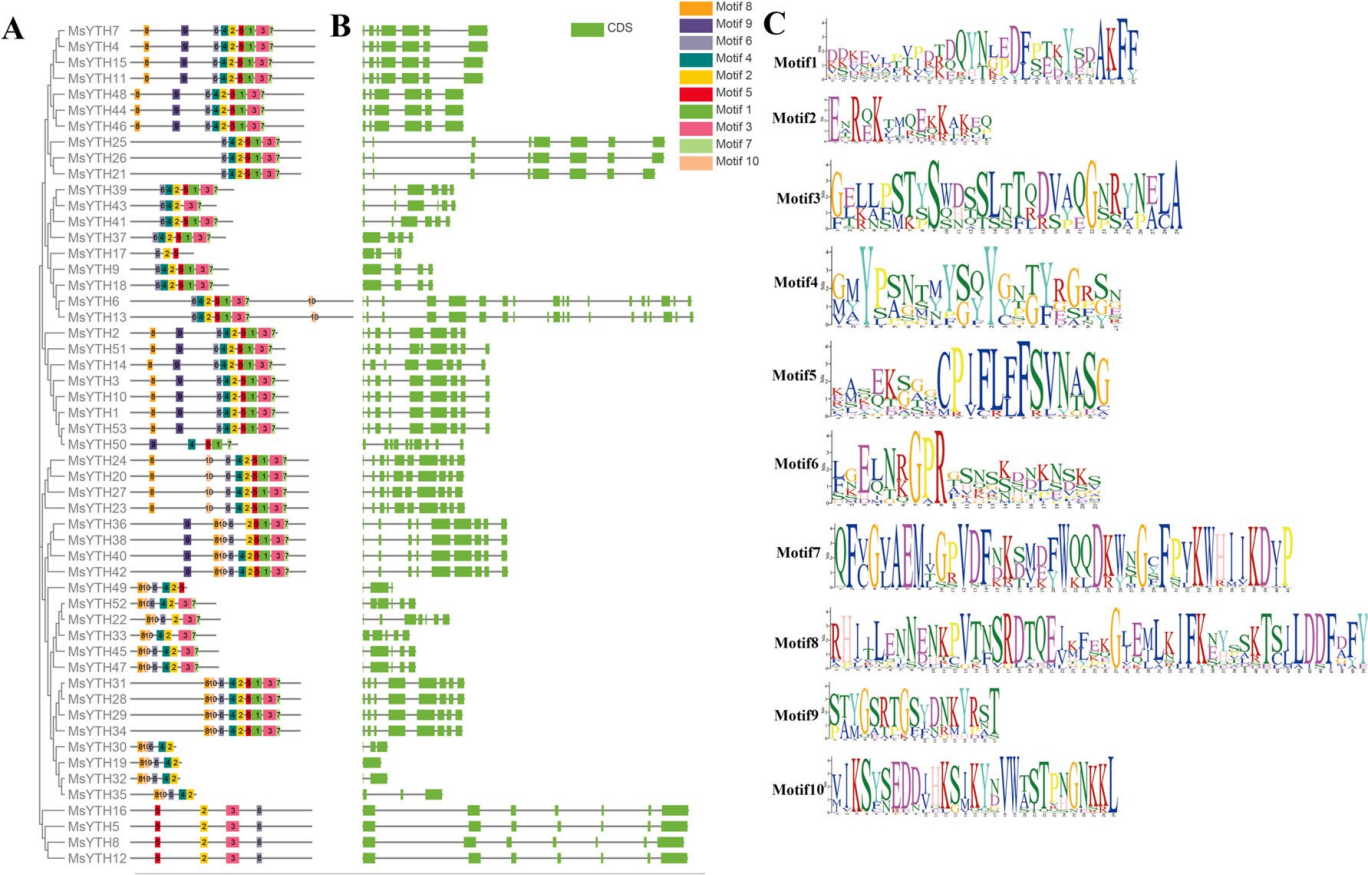
Conserved motif compositions and gene structure of MsYTH domain-containing proteins. **A** Graphical representation of the distributions of conserved motifs in MsYTH proteins. Each distinct motif is represented by a different color and associated number. **B** Schematic diagram of the exon/intron organization of MsYTH genes. Green boxes denote exons, and black lines indicate introns. **C** Detailed sequence logo analysis of the conserved motifs within the MsYTH proteins. Each motif is represented by a sequence logo with a corresponding number

The MEME Suite was employed to detect conserved motifs in MsYTH proteins. We found ten conserved motifs in YTH proteins, and the size of the motif varied widely with motif 8 being the largest and motif 2 being the smallest. Amino acid conservation per motif also varied widely. For example, AF, ERK, SGA, YY, F and GR were the most conserved amino acids in motifs 1 to 6, respectively, while motifs 7 and 8 had the most conserved amino acids (Fig. 3).

The proteins MsYTH5, MsYTH8, MsYTH12, and MsYTH16, which collectively constitute the YTHDCa subclass, each possess only four specific motifs—namely motifs 2, 3, 5, and 6. This reduced motif representation is considerably lower compared to the remaining members of the gene family. Notably, motifs 2 and 6 are the most common motifs, being present in 52 and 51 proteins, respectively. We also observed a noteworthy divergence between the MsYTHDC and MsYTHDF subfamilies. Specifically, in the MsYTHDC subfamily, we identified the presence of CCCH-type zinc fingers, a prominent structural motif crucial for protein-DNA and protein–protein interactions, characterized by the pattern C-X7-C-X5-C-X3-H. This motif is notably absent in the members of the MsYTHDF subfamily. The absence or presence of this zinc finger motif could signify distinct functional roles and regulatory mechanisms within these subfamilies.

Next, we assessed the variance in the quantity of exons and UTRs as shown in Fig. 3b. The number of exons and UTRs exhibited significant divergence across the examined gene samples. For instance, MsYTH19 presented only one exon while MsYTH6 and MsYTH13 exhibited as many as 19. It is hypothesized that the increased intron count within alfalfa YTHs could potentially bolster the repertoire of YTH proteins. Such an increase would, in theory, enable plants to embody more complex and varied biological functions.

### Protein 3D structure and conserved domain of MsYTH

**Fig. 4:**
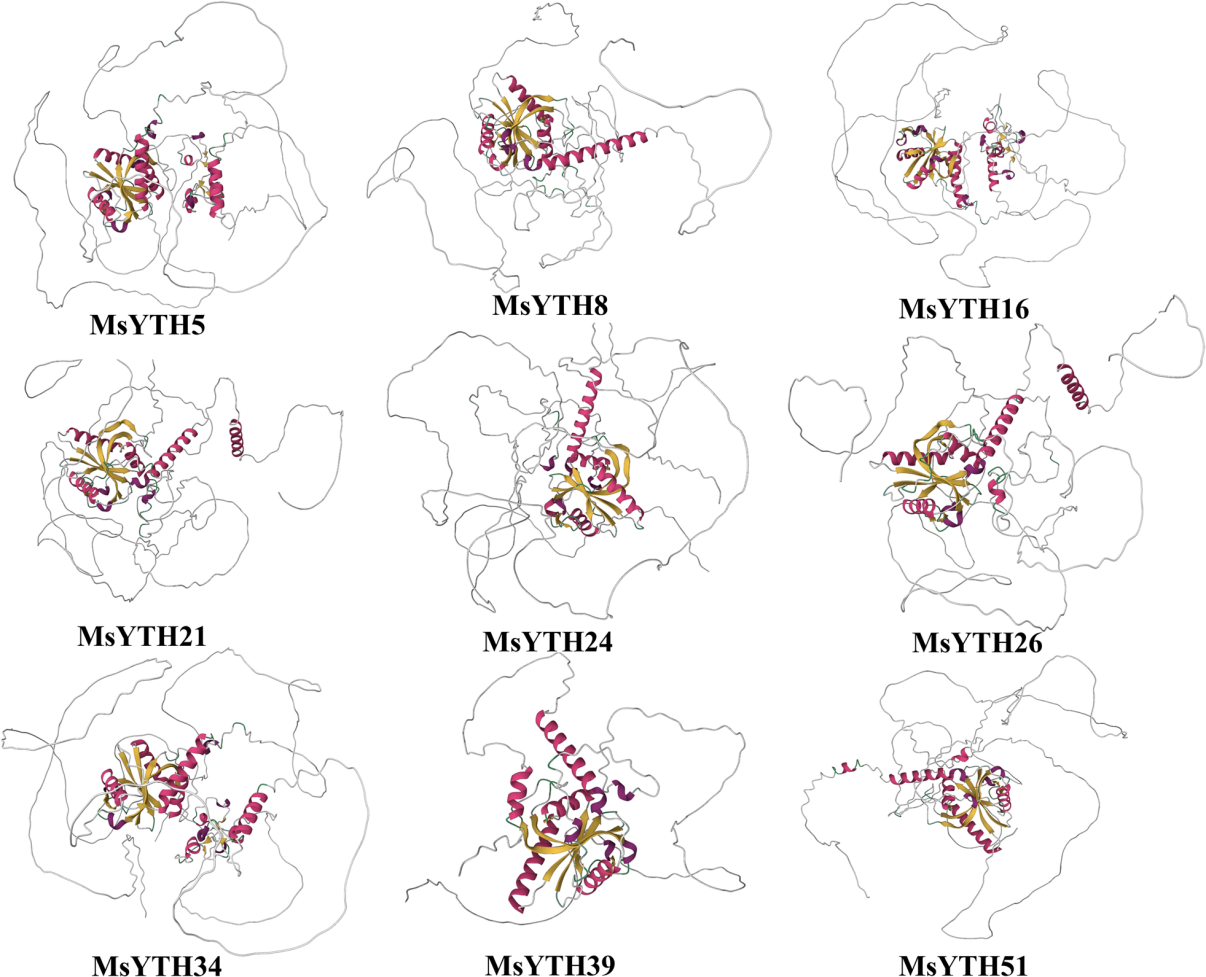
A three-dimensional schematic of the selected YTH protein structure predicted using AlphaFold2. The α-helix and β-sheet in the protein’s structure are represented by ochre and red colors, respectively, visualized using PyMOL2.5

To understand the protein structure, we used the default parameter for the structural creation of MsYTH proteins on a web platform (alphafold2: https://colab.research.google.com/github/sokrypton/ColabFold/blob/main/AlphaFold2.ipynb). Given the complexity, we strategically selected nine representative proteins from the four subclade within the gene family. These selected proteins cover the functional and structural diversity of the family, ensuring a comprehensive insight into the family’s characteristics. The general structure displays a globular fold with a four-stranded sheet center encircled by four helices, flanking areas on both sides, and a central core. This fundamental structure of a protein dictates its characteristics, whereas the higher structure controls its physiological activity [40]. The MsYTH models revealed that the binding sites of YTH domain-containing proteins are in the center of these folds, implying that YTH proteins in alfalfa include m6A binding sites and carry out their biological tasks by binding m6A (Fig. 4). The YTH domain is highly conserved, whereas the other regions don’t exhibit a fixed or ordered 3D structure. These areas, also known as intrinsically disordered proteins and intrinsically disordered regions (IDPs/IDRs), perform significant biological activities despite their lack of structural order. Their dynamic and disordered nature has been associated with key processes like enzyme catalysis and allosteric regulation. Moreover, they play crucial roles in essential physiological functions such as cell signaling and transcription [14, 46].

### Chromosome location, synteny, and gene duplication analysis

**Fig. 5:**
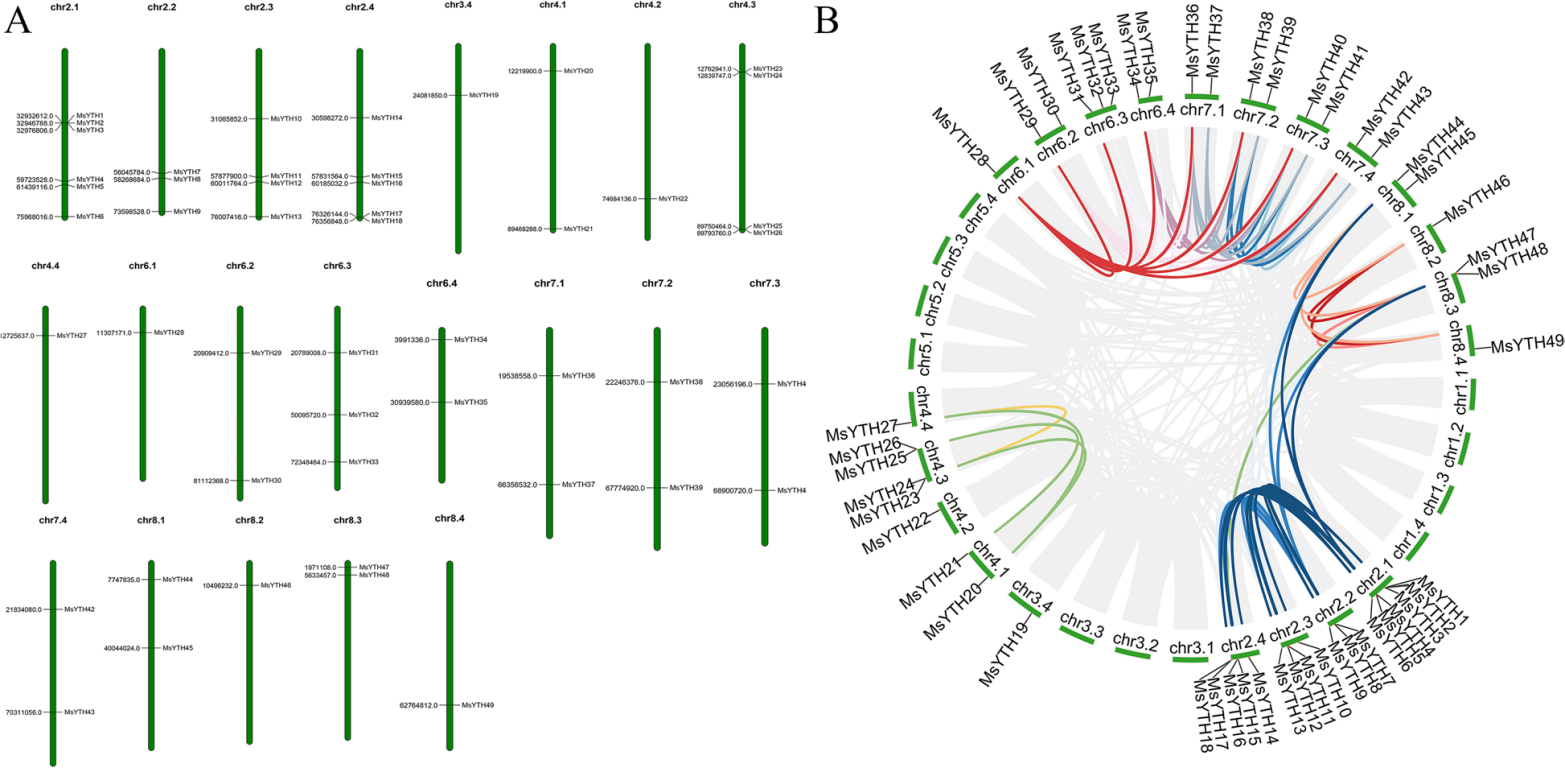
**A** Chromosomal distribution of MsYTH genes in alfalfa and **B** Syntenic analysis in alfalfa genome. The colored lines connecting the genes denote potential gene duplication pairs. The grey lines indicate syntenic blocks across the genome

In alfalfa, the 53 YTH genes were clustered across 21 chromosomes. The number of genes per chromosome varied widely. For instance, while chr2.1 had 12 genes, chr8.1 had only one gene. *MsYTH1/MsYTH2*, *MsYTH3*, and *MsYTH4* are members of the MsHDF subfamily and are found on chromosome 2.1. No relationship was observed between chromosomal length and the number of MsYTH genes. We further developed a comparative syntenic map between alfalfa and Arabidopsis to learn more about the evolutionary processes of the YTH family. On chr2.4, four genes *MsYTH14*, *MsYTH15*, *MsYTH17* and *MsYTH18* belonging to the YTHDFc subfamily are aggregated into one tandem duplication event area. Except for *MsYTH49* and *MsYTH19*, all *MsYTH* genes have a syntenic connection with Arabidopsis, indicating that these orthologous pairings may have existed before the divergence. The collinear gene pair associated with *MsYTH28* was not found in Arabidopsis, indicating that this gene might have been generated through gene duplication or segmental duplication events (Fig. 5).

In order to elucidate the evolutionary selection patterns within the YTH gene family in alfalfa, we quantitatively assessed the synonymous and non-synonymous substitution rates (Ka/Ks) of corresponding gene pairs (Table S3). Remarkably, only a minority of pairs (4 out of 72) displayed Ka/Ks values exceeding 1. This suggests that the majority of the YTH gene family has undergone negative selection, indicative of a relatively slow rate of evolutionary change.

### Cis-regulatory element analysis of MsYTH genes

**Fig. 6:**
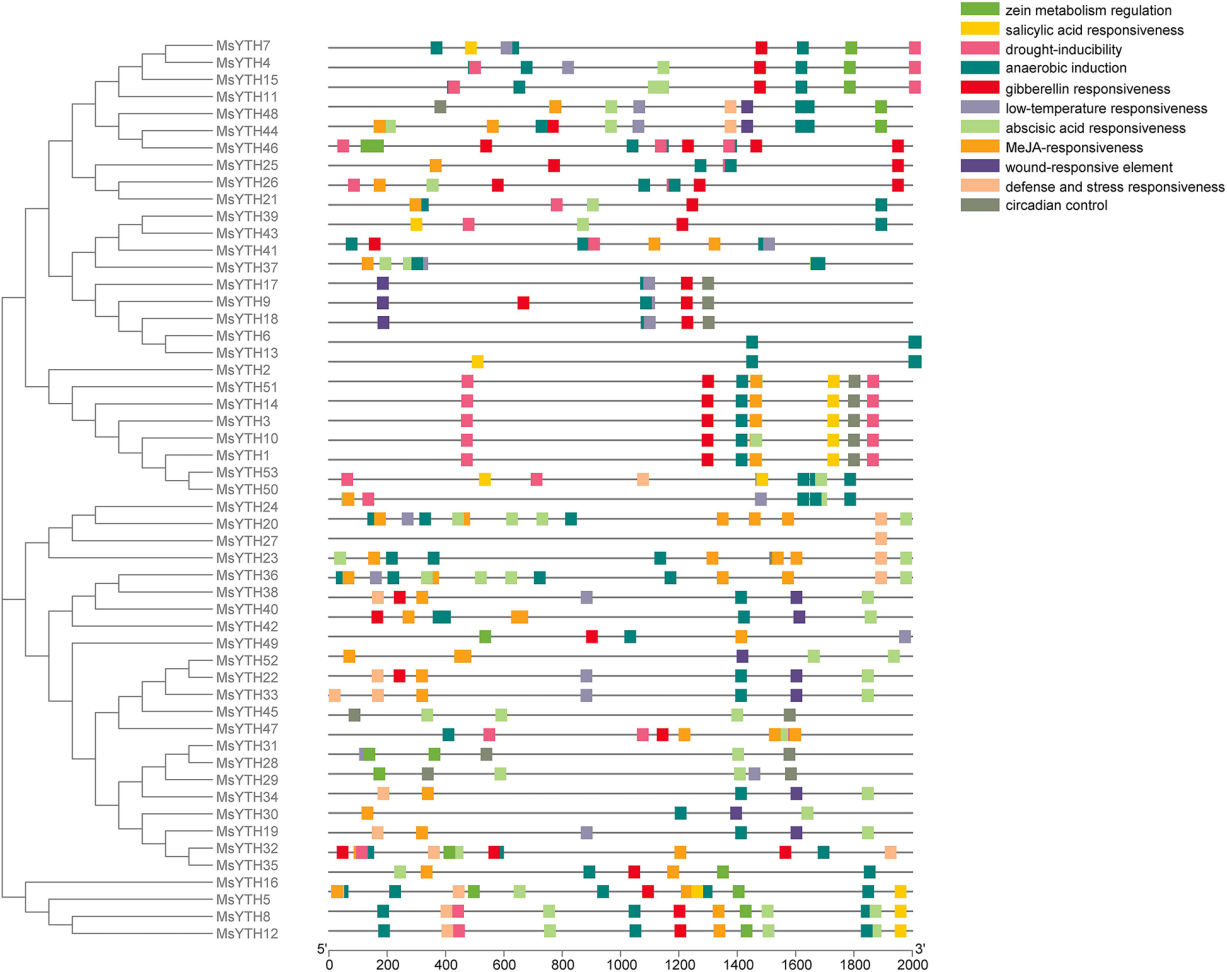
Identification of Predicted Cis-Acting Elements in Alfalfa Genes. Cis-acting elements were predicted using the PlantCARE database. The scale at the bottom allows inference of the distance upstream to the translation start site, providing insight into potential gene regulation locations

Multiple hormone-related elements were discovered in *MsYTH* promoters, including methyl jasmonate (MeJA) responsiveness (MsYTH2,4,6,7,9), abscisic acid responsiveness (MsYTH2-4,6–9), auxin responsiveness (MsYTH1,3–6,8), salicylic acid responsiveness (MsYTH4-6), and gibberellin responsiveness (MsYTH1,5,6). Furthermore, each promoter contained at least two categories of phytohormone-related elements. Multiple cis-acting regulatory elements implicated in abiotic stressors were also discovered, including drought-inducibility (*MsYTH1,3–7,9*), wound responsiveness (*MsYTH1,7,9*), defense and stress response (*MsYTH1,3,4,6*), and low-temperature responsiveness (*MsYTH3*) (Fig. 6). A circadian regulatory element was discovered in the promoters of *MsYTH1*, *MsYTH7*, and *MsYTH9*. *MsYTH7* and *MsYTH9* have the same cis-elements, suggesting that these two members may have comparable transcriptional regulation. These cis-acting regulatory elements found in *MsYTH* promoters suggested that the *MsYTH* family may be involved in controlling alfalfa plant response to hormone and abiotic stressors.

### RNA-seq expression profile

While attempting to examine the expression patterns of MsYTH genes in alfalfa, we encountered challenges in acquiring direct transcriptome data. As a workaround, we turned to the transcriptome data from CADL (Cultivated Alfalfa at the Diploid Level). Using this data, we investigated the homologs of 12 MsYTHs in CADL that exhibited the highest homology with those in alfalfa.

Our analysis revealed distinct expression patterns across nine different organs. In senescent leaves, the homologs of MsYTH7, 34 and 40 demonstrated low abundance, whereas the homologs of *MsYTH9*, *16*, and *17* showed high abundance. This expression pattern was consistent in young and mature leaves, with these genes clustering together. Notably, these homologs presented almost inverse expression patterns in the root, elongating stem, and post-elongating stem.

**Fig. 7:**
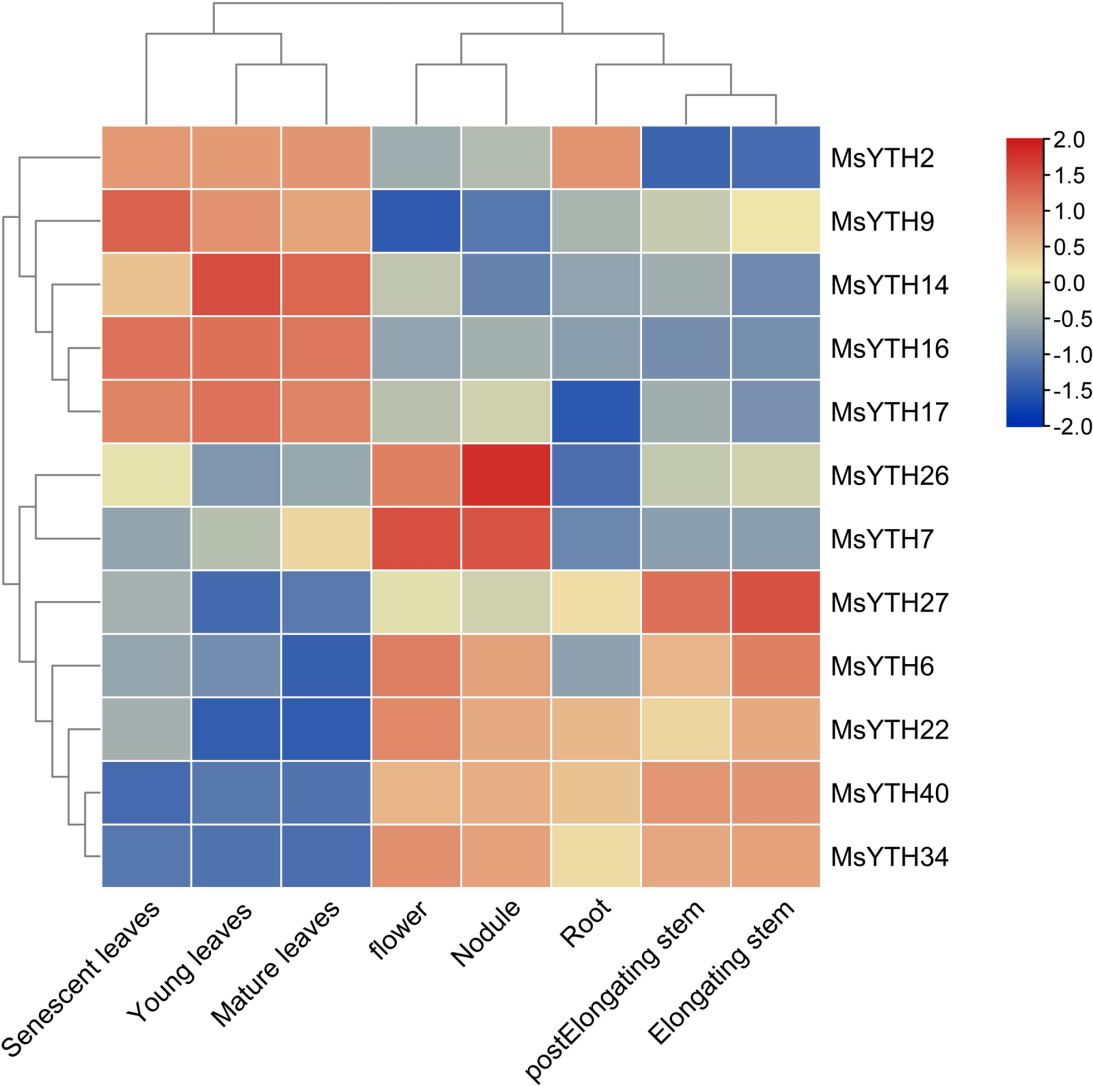
Expression analysis of 12 *MsYTH* gene homologs across various organs in CADL based on transcriptome data. FPKM values of genes were transformed by log10 and the heatmap was constructed by HemI software. Red represents high expression and green represents low expression level

The homologs of six genes, namely *MsYTH6*, *7*, *22*, *27*, *34* and *40* showed lower mRNA levels in senescent, young, and mature leaves as compared to their transcript accumulation in the post-elongation and elongation stem phases. The *MsYTH2* homolog exhibited the highest mRNA abundance in the leaf, whereas the homologs of *MsYTH9*, *16*, and *17* showed the highest expression levels during the early growth stages, as indicated in Fig. 7.

Moreover, the homologs of the *MsYTH* family displayed a variety of dynamic expression patterns throughout the flowering process. For instance, the *MsYTH16* homolog expression was consistently and significantly downregulated from post-flowering to maturity, while the *MsYTH7* homolog was upregulated exclusively during the leaf flower and nodule stages.

### Response of MsYTH genes to abiotic stresses and organ-specific expression patterns by qRT-PCR

When the expression of MsYTH in the root is compared to that in the flower, some MsYTHs are expressed significantly higher in the root, some MsYTHs are expressed lower int the root, and some MsYTHs show comparable expression in the. *MsYTH2*, *MsYTH14*, *MsYTH26*, *MsYTH37* and *MsYTH42* are all downregulated in the roots while maintaining high expression in the leaves. 12 of the genes (*MsYTH2*, *MsYTH7*, *MsYTH14*, *MsYTH16*, *MsYTH21*, *MsYTH27*, *MsYTH34*, *MsYTH37*, *MsYTH40*, *MsYTH42*, *MsYTH49*, and *MsYTH50*) are expressed significantly higher in flower when compared to that in roots.

**Fig. 8:**
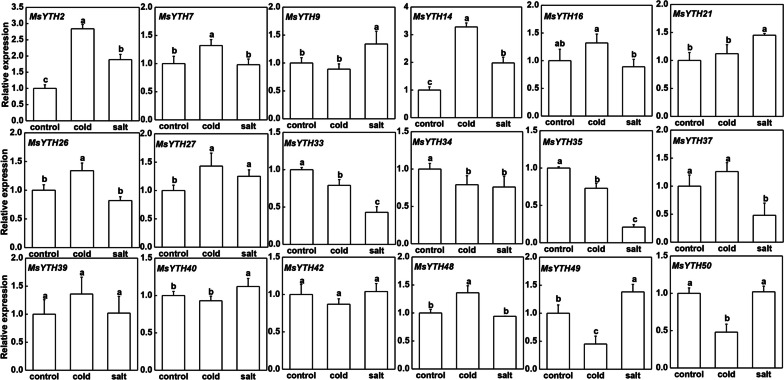
Bar plots showing the expression of MsYTH genes under cold and salt treatments. The x-axis represents the different treatments, while the y-axis represents the relative gene expression calculated using the 2^−∆∆Ct^ method. Each bar represents the mean ± standard deviation (SD) of three biological replicates. Lowercase letters above the bars indicate significant differences (*p* < 0.05) according to one-way ANOVA

Under abiotic stresses, a dramatic change in the expression of *MsYTH* genes in alfalfa is also observed (Fig. 8). For example, the highest upregulation of *MsYTH* under cold stress is observed in *MsYTH2* and *MsYTH14*, while under salinity conditions, MsYTH2 has an absolute advantage expression. This suggests that these genes may play important roles in both salt and cold tolerance in alfalfa. Most of the genes are upregulated more under cold conditions than under salt conditions. Overall, the findings shed light on the possible involvement of *MsYTH* proteins in alfalfa cold tolerance. The RNA-Seq data acquired from the CADL showed that the expression profiles of YTH genes were mainly consistent with our qRT-PCR results.

## Discussion

Based on the previous studies on the erasers and writers of m6A, it was uncovered that m6A modification has notable effects on the regulation of gene expression at the post-transcriptional level, and plays key roles in various physiological processes. To understand the regulatory mechanism of m6A, recognizing m6A sites by reader proteins is pivotal. These recognitions perform multiple functions of m6A modifications, determining the fate of their target RNAs and influencing physiological aspects [38].

The YTH (YT521-B homology) domain, a 100–150 amino acid long gene family, was the first identified m6A reader in mammals, including humans, and has been shown to be substantially conserved across the five kingdoms [1, 36]. Of the five kingdoms, plants have the most abundant YTH genes [31]. Among plants, Arabidopsis is the most studied species. In this plant, the protein reader m6A modification has been found to affect developmental timing and morphogenesis under abiotic stress [3]. The Arabidopsis genome contains 13 YTH domain proteins that may be split into two clades based on the C-terminal area, two members in the YTHDC clade (Evolutionarily Conserved C-Terminal region) and 11 members in the YTHDF clade [3].

The chromosomal-level genome of the tetraploid plant alfalfa, an important forage species, was not published until 2020, providing a significant advantage for understanding its evolution, genetics, and breeding. In this study, a total of 53 putative YTH domain-containing protein family genes were identified in alfalfa. This number exceeds that of all the 26 species reported by Ouyang [37], which include six Chlorophyta, one moss, one Lycopodiatae, four monocot species, and 15 eudicot species. The author in that study asserted that the number of YTH genes is not directly proportional to genome size. In our study, all 53 identified YTH domain-containing protein family genes were found to be distributed across 21 chromosomes in alfalfa. Notably, the distribution is heterogeneous, with some chromosomes harboring only one gene (chr8.4), while some others harboring six genes (chr2.1). This observation indicates the significance of both genome size and the occurrence of tandem and segmental duplications in shaping the YTH domain-containing protein family genes landscape of alfalfa.

The role of tandem and segmental duplication, recognized as crucial events in gene family expansion [7], emerges prominently in our study. For example, three *MsYTH1/MsYTH2/MsYTH3* genes cluster within 15 kb on chromosome 2.1, showing that both tandem and segmental duplication events were likely involved in the development of the *MsYTH* gene (Fig. 5). The pattern of functional redundancy among paralogs is similar to Arabidopsis, while segmental duplication dominated the evolution of YTH genes in rice [24] and cucumber [58]. These differences indicate potentially more complex regulatory mechanisms or functional redundancy within the YTH family in alfalfa, which require additional experimental validation in future studies.

The distribution of MsYTH within various groups displays a unique pattern when compared with Arabidopsis. For instance, the 13 YTH genes in Arabidopsis are broadly distributed across five groups, whereas in alfalfa, the majority of the YTH genes are primarily clustered within two groups, *YTHDFa* (22 genes) and *YTHDFc* (24 genes). Our study did not identify any alfalfa genes that belong to YTHDCb group. This gene distribution pattern deviates significantly from the typical configurations reported in the 26 species analyzed by Ouyang et al. [37], where no species had more than 20 YTH genes. Conversely, this distribution aligns more closely with the pattern reported in wheat, which possesses 39 YTH genes [45]. The distinct concentration of YTH genes in the YTHDFa and YTHDFc groups in alfalfa suggests possible functional redundancy within these specific gene subsets. This conjecture supports the theory of gene duplication and diversification being a critical evolutionary strategy in plants.

Members of each YTH subfamily have completely distinct gene architectures in terms of the size and organization of exons and introns (Fig. 3), showing that these genes developed separately. Members of the YTHDC subfamily have comparable gene structures, suggesting that these genes are created through gene duplication (Fig. 3a).

A search utilizing the SMART algorithm revealed the existence of a typical functioning YTH domain in each MsYTH protein. YTH domain are generally found near the C-terminus in the alfalfa YTHDF group but found near the N-terminus in alfalfa YTHDFc subgroup. In the alfalfa YTHDCa subgroup, YTH domains are located at the midway point. The YTH domain and the motifs are distributed similarly among members of each subfamily or subclade, indicating that they are highly conserved. Detailed motif analysis further revealed the evolutionary connections between members of the MsYTH family. Certain amino acid-rich motifs in the aligned protein sequence, such as the leucine-rich repeat domain, might be connected to salt resistance (Fig. 3c). Motifs 1–4 of the YTHDFa subclades were found to be identical to those reported in previous research on YTH domains [45]. In addition to the YTH domain, we have also discovered the presence of CCCH-type zinc finger motifs exclusively within the YTHDC clade of alfalfa YTH genes (Fig. 2). This finding aligns with the pattern observed in wheat YTH proteins, further highlighting the evolutionary conservation of this motif within the YTHDC subfamily. The CCCH-type zinc finger motifs have been associated with RNA-binding activities and have been implicated in post-transcriptional regulation processes [18]. The identification of these motifs within the YTHDC clade suggests potential functional implications related to RNA-binding and post-transcriptional regulation in this specific subclades of YTH genes in both alfalfa and wheat.

Looking further into the amino acids of the motifs, the aromatic cage pocket in the YTH domain of YTHDF and YTHDC functions to bind the m6A residue [17]. This positively charged pocket is created by the side chains of three amino acids: tryptophan (W411), tryptophan (W465), and tryptophan (W470) in human YTHDF1 and tryptophan (W377), tryptophan (W428), and leucine (L439) in human YTHDC1 [25, 38]. Specifically, proteins encoded by MsYTHDF genes incorporate a single domain structure, while those corresponding to *MsYTH8*, *MsYTH12*, *MsYTH16* are identified as members of the *MsYTHDC* subfamily, exhibit CCCH-type zinc finger repeats at their N-termini. It has also been observed that the predicted aromatic cage pocket that binds the m6A residue of *MsYTHC* primarily consists of a sequence of three tryptophan residues (WWW). Conversely, the binding pocket was composed of two highly conserved tryptophan residues and either one tryptophan residue (WWW) or tyrosine (WWY) in MsYTHDF. This pattern aligns with those observed in wheat, suggesting a potential divergence in m6A binding between plant and animal YTHDCs.

In animals, the YTH domain is centrally located in YTHDCa, while it is found at the C-terminus in YTHDCb. We identified similar variations in plant species. However, in plant’s YTHDCs, all YTH domains are observed to be centrally located, akin to the positioning in animal YTHDCa. This may imply that the molecular mechanisms in plant YTHDCs could parallel those in animal YTHDCa.

A study of the binding affinity of YTHDF1 and YTHDC1 for m6A indicated that the asparagine (N367) in YTHDC1 forms a stronger hydrogen bond with N1 of m6A than the comparable aspartic acid (D401) residue in YTHDFa [20]. We observed that YTHDFa, YTHDFb, and YTHDFc proteins use aspartic acid to form a hydrogen bond with N1 of m6A; however, aspartic acid has been replaced by asparagine in six YTHDFb, three YTHDFc, and three YTHDC proteins. Moreover, in three YTHDFb proteins, histidine replaceds the usual residue at the corresponding location, a pattern not identified in YTH proteins of other plant species. The N-termini of YTHDF proteins have low-complexity areas, including Y/P/Q-rich regions; however, the Y/P/Q-rich regions of YTHDCs were found between the zinc finger repeat (YTH1 superfamily domain) and the YTH domain.

Information about the promoter sequence may provide insight into the gene’s activities. In this study, it was observed that light responsiveness had the most cis-regulatory elements among all the genes, suggesting a photoperiodism-related function. The second most prevalent cis-regulatory element is linked to stress-signaling hormones such as MeJA, salicylic acid, abscisic acid, and gibberellins. Other notable features included zein metabolism and regulation, circadian control, MYB binding, and anaerobic control.

**Fig. 9:**
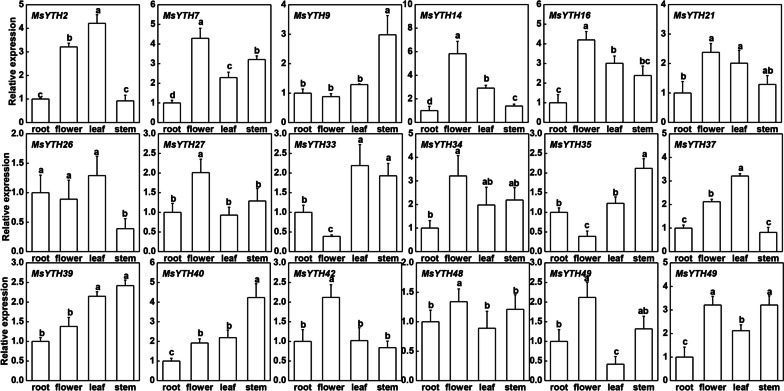
Bar plots showing the expression of MsYTH genes in various alfalfa organs. The x-axis represents the different organs, while the y-axis shows the relative gene expression calculated using the 2^−∆∆Ct^ method. Each bar represents the mean ± standard deviation (SD) of three biological replicates. Lowercase letters above the bars indicate significant differences (*p* < 0.05) according to one-way ANOVA

YTH genes were expressed in all the investigated organs and showed diverse expression patterns. Several members of the YTH family have organ-specific expression patterns indicating that they may have various potential functions in different organs (Fig. 9). In line with this, it has been noted in previous research that distinct gene expression profiles in different organs can provide insights into gene functions, especially in relation to plant development and morphology [24].

YTH-containing genes in many species can transcriptionally respond to abiotic stress, such as excessive salinity, drought, heat, cold, and polyethylene glycol stress [24, 58]. In this study, several stress-related cis-elements have been discovered in the upstream regions of the promoters of YTH members, including heat stress-responsive elements, drought-responsive elements, defense- and stress-responsive elements (TC-rich repeats), anaerobic induction elements, and low temperature-responsive elements, all of which respond to external environmental stresses [24].

The qRT-PCR results revealed distinct expression patterns for various genes in response to both cold and salt treatments. For example, *MsYTH2* and *MsYTH14* exhibited significant up-regulation under both stress conditions, implying a potential shared regulatory mechanism in addressing these environmental challenges. In contrast, both *MsYTH33* and *MsYTH35* consistently displayed down-regulation in response to cold and salt stress. *MsYTH9* showed no significant difference in gene expression under cold stress but demonstrated up-regulation in response to salt stress. Notably, *AtYHT3* encoding *AtCPSF30*, which is the homologous to *MsYTH9*, has been proven to enhance tolerance to oxidative stress by influencing poly(A) site selection and mRNA profiles [24]. This suggests the possibility of similar stress tolerance mechanisms among YTH genes in alfalfa.

## Conclusion

In conclusion, our study identified and characterized 53 putative YTH domain-containing genes in alfalfa, highlighting their potential roles in the regulating various physiological processes, particularly in response to abiotic stress conditions. Expression profile analysis revealed that specific genes, such as *MsYTH2*, exhibited enhanced expression in response to salt and cold treatments, suggesting their possible involvement in managing abiotic stress responses. Additionally, the distinct motif distributions and domain architectures within the YTH gene family were observed. Our findings shed light on the biological functions of YTH genes in alfalfa and provide valuable insights for future genetic enhancement strategies aimed at improving salt and cold tolerance in this important forage crop.

### Supplementary Information

**Additional file 1: Table S1.** Sequences of all the MsYTH protein.

**Additional file 2: Table S2.** List of Primers used for qRT-PCR genes.

**Additional file 3: Table S3.** Synonymous and Non-Synonymous substitution rates (Ka/Ks) for corresponding gene pairs within the alfalfa YTH gene family.

## Data Availability

The whole alfalfa genome sequence information was obtained from the database (https://figshare.com/projects/whole_genome_sequencing_and_assembly_of_Medicago_sativa/66380). Alfalfa seeds used for experimental is given by Dr. Dong from Guizhou University. The datasets supporting the conclusions of this article are included in the article and its Additional files.
